# Early feeding practices and associated factors in Sudan: a cross-sectional analysis from multiple Indicator cluster survey

**DOI:** 10.1186/s13006-020-00288-7

**Published:** 2020-05-14

**Authors:** Manar E. Abdel-Rahman, Asmaa El-Heneidy, Lenka Benova, Laura Oakley

**Affiliations:** 1 Department of Public Health, College of Health Sciences, QU Health, Qatar University, Doha, Qatar; 2 School of Medicine, Griffith University, Gold Coast, Queensland Australia; 3 Department of Public Health, Institute of Tropical Medicine, Antwerp, Belgium; 4 Department of Non-communicable Disease Epidemiology, London School of Hygiene and Tropical Medicine, London, UK; 5 Centre for Fertility and Health, Norwegian Institute of Public Health, Oslo, Norway

**Keywords:** Breastfeeding, Early initiation, Timely initiation, Prelacteal feeding, Associated factors, Determinants Sudan

## Abstract

**Background:**

In efforts to reduce neonatal mortality, the World Health Organization (WHO) has included breastfeeding among its recommended packages of interventions. Early initiation of breastfeeding and avoidance of prelacteal feeding are key contributors to optimal feeding practices. This study aims to assess the prevalence and associated factors of early breastfeeding practices in Sudan.

**Methods:**

This study utilises the cross-sectional nationally-representative Sudan Multiple Indicator Cluster Survey (MICS) conducted in 2014. The sample includes women who had a live birth in the two years before the survey and their self-report on early breastfeeding practices, namely early initiation and prelacteal feeding. Percentages of these early breastfeeding practices indicators were estimated accounting for the complex survey design. Multivariable logistic regression analyses were used to examine the factors associated with these outcomes.

**Results:**

Of 5622 mothers, 69% initiated breastfeeding within one hour of birth, 72% avoided prelacteal feeding in the first three days after birth, and 51% met the criteria for both (i.e. practised optimal early feeding practice). Optimal early feeding varied across regions of Sudan. Birth by caesarean section (Adjusted Odds Ratio [AOR] 0.34; 95% CI 0.25, 0.47) and at a health facility (AOR 0.75; 95% CI 0.60, 0.94) were negatively associated with optimal early feeding practice. Mothers with secondary education (AOR 1.62; 95% CI 1.30, 2.02), those who desired their pregnancy at the time (AOR 1.31; 95% CI 1.08, 1.60), those who were assisted by a skilled birth attendant at birth (AOR 1.48; 95% CI 1.19, 1.83), and those who gave birth to female infants (AOR 1.16; 95% CI 1.02, 1.33) had higher odds of use optimal early feeding practice. Similarly, the odds of optimal early feeding increased with parity and maternal age.

**Conclusions:**

Only half of Sudanese mothers practised optimal early feeding practice, with important differences between regions in the country. Early feeding practices in Sudan are associated with various maternal, child and community level factors. The findings suggest the need to develop breastfeeding promotion programs with consideration of regional variations and healthcare system interventions.

## Background

Despite a substantial reduction in child mortality from 12.7 million in 1990 to 6.3 million in 2017, the proportion of child deaths attributable to neonatal mortality is increasing [1]. Globally, neonatal mortality represents approximately 40% of under-5 deaths in 2017 [1]. In efforts to reduce neonatal mortality, the World Health Organization (WHO) has included breastfeeding among its recommended packages of interventions [2]. Breastfeeding is linked to lower neonatal and child mortality, and prevention of childhood morbidities such as diarrhoea and respiratory infection. Additionally, there is some evidence that it is linked to a reduction in the risk of obesity and diabetes later in life [3].

Early or timely initiation of breastfeeding, ideally within the first hour after birth, is vital for both the mother and the infant. Despite the well documented health benefits of early initiation for maternal health [4, 5] and infant survival [6], the percentage of infants who are breastfed within the first hour after birth remains suboptimal in many countries, [3] ranging from 18 to 98% with an average of 58% [7]. In a recent meta-analysis, Alzaheb found that only 34% of newborns in the Middle East received breastmilk within one hour of birth [8].

The introduction of prelacteal feeds (i.e. any food or drink other than breast milk) postpones the initiation of breastfeeding [9], potentially reducing the immunological benefits a newborn obtains and increasing his/her susceptibility to infection [10]. Prelacteal feeding can also be a direct cause of diseases by exposing infants to contaminated feeds, utensils, water, or hands. It may also affect neonatal health by disrupting the preparation of the gastrointestinal tract [11]. Usually driven by belief and traditions [12, 13], prelacteal feeding is common in many low- and middle-income countries. A recent study found that 32% percent of infants in 22 sub-Saharan African countries received prelacteal feeding; with country estimates ranging from 3% (Malawi) to 67% (Côte d’Ivoire) [14].

Early feeding practices of infants are studied through two indicators developed by the WHO: early initiation within one hour and exclusive breastfeeding [15]. In the first three days after birth, an infant may be regarded as exclusively breastfed if he/she did not have any prelacteal feeds. Oakley et al. proposed an optimal early feeding indicator which captures both early initiation and no prelacteal feeding in the first three days after birth [16].

A number of factors are associated with early breastfeeding practices, such as area of residence, region, socio-economic status, paternal education, maternal age at marriage, desire for birth, antenatal care, place of birth, attendance of birth by skilled personnel, mode of delivery, perceived size of infant, sex of infant, and birth order [16–19]. Some of these factors have also been identified as being associated with prelacteal feeding [9, 13, 14]. It is apparent from the studies that early breastfeeding indicators and their associated factors vary between and within countries.

Sudan is one of the largest countries in Africa with an area of 1.886 million km^2^, a population of 40.53 million and diverse ethnic blend of Africans and Afro-Arabs. It is one of the least developed countries according to the United Nations classification with GDP per capita of 977.3 US dollars in 2018 [20, 21]. Levels of child, infant and neonatal mortality in Sudan are among the highest in the world [22]. The Baby-Friendly Hospital Initiative (BFHI) promoting successful breastfeeding was first implemented in Sudan in 1995, where 71% of facilities were ever designated, and 21% were designated in 2012–2017 [23]. In 2013, only three facilities were available [24].

There is a lack of research on early feeding practices in Sudan, with previous studies with small samples and community-based [17, 25]. The main objective of this study is to assess national community-level, household level, parental and child-related factors associated with early initiation of breastfeeding, no prelacteal feed and optimal feeding using data from a recent nationally representative survey in Sudan. This research will contribute to knowledge with the potential to feed into the country’s public health policies and interventions.

## Methods

### Data sources

The 2014 Sudan Multiple Indicator Cluster Survey (MICS) is a representative survey designed to provide national-level data as well as state-level estimates for maternal and child health in the 18 states in Sudan. Sudan Central Bureau of Statistics (CBS) in collaboration with the Ministry of Health, conducted this survey as part of the global MICS programme [26]. The website of the MICS programme makes the anonymised survey dataset freely available.

### Study design and data collection

This study utilises cross-sectional secondary data from the 2014 Sudan MICS. This survey utilised an updated 2008 population census and randomly selected 40 enumeration areas in each State. The survey design employed two-stage cluster sampling after stratifying by urban and rural areas within each State. In the first stage, within each stratum, enumeration areas were selected with probability proportional to size. In the second stage, 25 households were selected in each enumeration area. Paper-based questionnaires were used for data collection using the Arabic language. Data were collected using three questionnaires, collecting information at the household level, on all eligible ever-married women aged 15–49 years and on all eligible children under-5 years of age. The survey sampled 18,000 households of which 16,801 were interviewed; 18,302 eligible women and 14,081 mothers/caretakers of children under-5 were interviewed. Detailed reports on the survey design, methods and findings of the survey can be found on the MICS website [27].

### Study sample

Data was collected from the three MIC questionnaires: breastfeeding information from the women questionnaire; child’s birth order from the children under-5 questionnaire and sociodemographic data from the household questionnaire, respectively. Analysis for this study was restricted to data from mothers with last-born children in the two years preceding the survey. The total sample size was 5622 mother-baby pairs.

### Definition of variables

#### Outcome variables

To address the study objectives, three early feeding indicators were the outcome variables: (1) early initiation within one hour of birth, (2) no prelacteal feeding in the first three days after birth, and (3) optimal feeding (1 and 2). In the women questionnaire, mothers were asked: “How long after birth did you first put (*child’s name*) to the breast?” Women responded by providing the number of hours or days. The outcome variable “early initiation” was defined by a binary variable reflecting the initiation of breastfeeding within one hour of birth (Yes/No). “No prelacteal feeding”, a binary (Yes/No) outcome variable, reflected the negative response to the question: “In the first three days after delivery, was (*child’s name*) given anything to drink other than breast milk?”. The “Optimal early feeding”, a binary outcome variable (Yes/No), was designed to capture children who both had early initiation within one hour of birth and did not have any prelacteal feeding in the first three days after delivery [16].

#### Explanatory variables

The selection of potential factors was informed by the literature [14, 18, 19, 28, 29] and grouped into community, household, parental and child-related variables. Community-level variables included: area and region of residence. For this study, the 18 States in the country were grouped into the original six regions formed at the country’s independence in 1956. Household variable consisted of the wealth index that was designed to capture underlying long-term wealth based on ownership of durable goods, housing characteristics and basic services using principal component analysis to rank households into wealth quintiles from poorest to richest [30]. Parental variables included maternal age at the time of the survey in years, maternal age at marriage in years, mother’s and father’s education, whether pregnancy was planned or not, number of times mother received antenatal care, place of birth, birth assisted by any skilled attendant (doctor, nurse/ midwife, health visitor, medical assistant), birth by caesarean section. Child-related variables included sex, birth order and how the mother perceived her child’s size at birth (larger than average, average, smaller than average).

### Statistical analysis

Percentages and 95% confidence intervals (95% CI) of the three breastfeeding outcome indicators were reported; overall and by explanatory variables. Logistic regression analyses were used to model the odds of the outcome variables. Crude logistic regression analyses were performed as initial steps of qualifying factors to be included in the multivariable logistic regression analyses. Potential factors with *p* - values < 0*.*25 were considered to develop an initial reduced model [31]. Using the adjusted Wald test, variables that tested insignificant (with *p* - values > 0.05) were then eliminated from this model. Confounding was assessed; variables were included in the model if their coefficients changed by 20% or more. Identified a priori as a confounder, maternal age was included in all models. F-adjusted mean residual goodness-of-fit tests were used to assess any evidence to lack of fit of the final model [31]. Odds ratios (OR), adjusted odds ratios (AOR) and their 95% CI from logistic regression analyses were reported. Stata version 15.0 was used for all analyses. *Svy* commands using Taylor linearization for the variance estimation were used to account for the complex survey design using assigned weights, primary sampling units (clusters) and strata. The sample weights were the inverse of the probability that the observation was included because of the sampling design. Weighted sample sizes and percentage are reported unless otherwise indicated. Missing values in the outcome variables were handles similarly as in MICS methodology in order to produce similar indicators as in previously published reports [27]. Multiple imputation was used to account for missing data in the predictors.

## Results

### Characteristics of the sample

A total of 5622 observations of mothers/last-born child pairs within two years preceding the survey were included in this study. Around 96% of mothers reported ever-breastfeeding their child. The majority of the sample resided in rural areas (73.5%), in Darfur (27.9%), and Central (29.1%) regions (Table 1). Half of the mothers were aged 25–34 years, three-quarters had primary, or no education and only 7.3% completed higher education. Only 9.1% of births were given birth by caesarean section, mostly born at home (71.3%) and attended by skilled health personnel (77.7%).

**Table 1:** Characteristics of the study sample and prevalence of early feeding practices

	n (%)	Early breastfeeding^**^	Avoiding prelacteal feeding^***^	Optimal early feeding
Overall	5622 (100.0)	68.7 (66.7,70.7)	71.7 (69.6,73.8)	51.0 (48.9,53.1)
Community-level variables				
**Area of residence**				
Urban	1488 (26.5)	71.0 (67.6,74.2)	69.9 (66.5,73.1)	50.3 (46.8,53.7)
Rural	4134 (73.5)	67.9 (65.4,70.3)	72.4 (69.6,75.0)	51.3 (48.6,53.9)
**Region of residence**				
Khartoum	684 (12.2)	73.7 (68.2,78.5)	64.1 (58.5,69.4)	47.9 (41.7,54.1)
Northern	244 (4.3)	59.9 (54.4,65.2)	56.2 (49.7,62.5)	33.4 (28.1,39.3)
Eastern	598 (10.6)	83.2 (79.1,86.7)	77.7 (73.9,81.1)	65.2 (60.6,69.4)
Central	1638 (29.1)	69.9 (65.8,73.7)	65.8 (60.7,70.5)	47.4 (43.0,52.0)
Kordofan	888 (15.8)	64.5 (58.3,70.3)	73.4 (68.6,77.7)	51.0 (45.5,56.5)
Darfur	1570 (27.9)	63.5 (60.1,66.9)	80.5 (76.9,83.6)	53.4 (50.1,56.7)
Household-level variables				
**Wealth index quintile**				
Poorest	1251 (22.2)	62.1 (57.3,66.7)	76.1 (71.6,80.0)	51.3 (46.7,55.8)
Second	1232 (21.9)	69.8 (66.5,73.0)	77.7 (74.6,80.6)	55.9 (52.5,59.3)
Middle	1192 (21.2)	73.1 (69.5,76.5)	69.6 (65.9,73.2)	52.6 (48.7,56.5)
Fourth	1096 (19.5)	68.2 (64.3,71.9)	65.7 (59.8,71.1)	45.4 (40.1,50.8)
Richest	851 (15.1)	71.3 (66.5,75.6)	67.5 (62.3,72.2)	48.4 (43.5,53.3)
Parental variables				
**Maternal age in years**				
15–24	1515 (26.9)	66.3 (63.2,69.2)	69.4 (65.7,72.9)	47.1 (43.4,50.9)
25–34	2802 (49.8)	70.5 (67.8,73.0)	72.7 (70.2,75.0)	52.8 (50.2,55.4)
35+	1305 (23.2)	67.8 (64.5,70.9)	72.4 (68.4,76.1)	51.7 (48.0,55.3)
**Maternal age at marriage in years**				
< 15	918 (16.3)	64.7 (60.4,68.8)	68.9 (64.2,73.3)	46.3 (42.0,50.6)
15–24	4189 (74.5)	69.4 (67.0,71.7)	72.3 (69.9,74.6)	52 (49.5,54.4)
25+	514 (9.2)	70.0 (64.3,75.1)	72.1 (65.9,77.6)	51.8 (46.2,57.3)
**Mother’s education**				
None	2247 (40.0)	68.0 (65.0,70.8)	72.3 (69.4,75.1)	51.2 (48.3,54.1)
Primary	2022 (36.0)	67.5 (63.7,71.0)	70.6 (66.7,74.2)	49.7 (46.1,53.4)
Secondary	942 (16.8)	73.1 (69.1,76.8)	74.6 (70.5,78.4)	54.7 (50.3,59.0)
Higher	410 (7.3)	68.8 (61.4,75.3)	67.5 (59.9,74.2)	47.3 (40.3,54.5)
**Father’s education**				
None	1880 (33.9)	68.2 (64.9,71.3)	71.7 (68.4,74.8)	51.2 (47.7,54.7)
Primary	1642 (29.6)	69.8 (66.4,73.0)	71.3 (67.3,75.0)	52.4 (48.6,56.2)
Secondary	968 (17.5)	72.9 (69.1,76.4)	71.3 (67.6,74.7)	53.8 (49.4,58.2)
Higher	320 (5.8)	75.8 (69.8,80.9)	68.2 (61.0,74.7)	53.7 (46.9,60.3)
Father not in household	692 (12.5)	63.0 (57.7,68.0)	72.7 (67.6,77.3)	44.7 (39.8,49.7)
Missing/dk	38 (0.7)			
**Planned pregnancy**				
No	1087 (19.3)	67.3 (62.6,71.7)	64.6 (60.3,68.6)	46.2 (42.3,50.1)
Yes	4491 (79.9)	69.6 (67.4,71.7)	73.2 (70.7,75.6)	52.6 (50.1,55.0)
Missing	44 (0.8)			
**Number of antenatal care visits**				
0	1080 (19.2)	67.3 (62.3,71.8)	73.1 (68.1,77.6)	53.3 (48.7,57.8)
1–3	1596 (28.4)	71.0 (67.6,74.1)	71.4 (68.5,74.2)	51.4 (47.9,54.9)
4+	2852 (50.7)	68.7 (66.2,71.0)	70.9 (67.8,73.8)	50.3 (47.5,53.1)
Missing	94 (1.7)			
**Place of birth**				
Home	4006 (71.3)	71.5 (69.1,73.8)	73.7 (71.5,75.7)	54.8 (52.5,57.1)
Health facility	1559 (27.7)	63.5 (60.2,66.6)	65.8 (61.6,69.7)	42.5 (38.9,46.1)
Missing	57 (1.0)			
**Birth assisted by skilled attendant**				
No	1252 (22.3)	63.6 (59.4,67.5)	72.3 (68.1,76.1)	48.3 (44.3,52.3)
Yes	4370 (77.7)	70.2 (68.0,72.3)	71.6 (69.1,73.9)	51.8 (49.3,54.2)
**Birth by caesarean section**				
No	5110 (90.9)	71.3 (69.2,73.2)	73.1 (70.9,75.1)	53.4 (51.2,55.6)
Yes	511 (9.1)	43.3 (37.5,49.3)	58.4 (52.4,64.2)	26.8 (21.6,32.7)
Child variables				
**Child sex**				
Male	2809 (50.0)	68.4 (65.9,70.9)	70.3 (67.6,72.8)	49.8 (46.9,52.6)
Female	2734 (48.6)	70.1 (67.8,72.4)	72.8 (70.2,75.2)	53.0 (50.6,55.4)
Missing	79 (1.4)			
**Birth order**				
1st	1984 (35.3)	66.2 (63.0,69.2)	69.8 (66.9,72.6)	47.4 (44.1,50.7)
2nd	2970 (52.8)	71.2 (68.6,73.6)	72.5 (69.9,74.9)	53.5 (50.8,56.1)
3rd or higher	588 (10.5)	70.4 (65.0,75.2)	72.3 (67.1,76.8)	54.3 (49.0,59.5)
Missing	79 (1.4)			
**Perceived size at birth**				
Larger than average	727 (12.9)	65.8 (61.4,69.9)	72.8 (67.6,77.5)	48.9 (43.7,54.2)
Average	2893 (51.5)	70.9 (68.4,73.3)	72.2 (69.7,74.6)	52.7 (50.0,55.4)
Smaller than average	1903 (33.9)	68.2 (64.6,71.6)	69.9 (66.7,72.9)	50.3 (46.7,53.8)
DK or missing	99 (1.8)			

** 257 unweighted missing cases (4.38%) were assumed not breastfed with one hour

*** 249 unweighted missing cases (4.52%) were assumed not given any prelacteal feed

### Early initiation of breastfeeding

Early initiation of breastfeeding was reported by 68.7% (95% CI 66.7, 70.7) of women (Table 1). It was least prevalent in the Northern region 59.9% (95% CI 54.4, 65.2) and most prevalent in Eastern region 83.2% (95% CI 79.1, 86.7).

Univariate logistic regression analyses revealed that early initiation was significantly associated with region of residence, household wealth, father’s education, place of birth, birth assisted by a skilled attendant, birth by caesarean section, and birth order (Supplementary Table S1).

Compared to the capital city of Khartoum, the adjusted odds of early initiation was lower among mothers who were residents in all other regions; the Eastern region was an exception (Table 2). The adjusted odds of early initiation among mothers in this region was 1.79 higher compared to Khartoum (AOR 1.79; 95% CI 1.21, 2.64; *p* = 0.004). These odds were significantly higher with increasing father’s education. This result was similar for mother’s education up to secondary level where the adjusted odds of early initiation decreased for women with higher education compared to those with no education. The adjusted odds of early initiation was 34% higher among mothers whose births were assisted by a skilled attendant compared to unassisted mothers (AOR 1.34; 95% CI 1.06, 1.68; *p* = 0.014). Compared to mothers who gave birth by vaginal delivery, those who gave birth by caesarean section had 78% lower odds of commencing breastfeeding within the first hour after birth (AOR 0.22; 95% CI 0.17, 0.27; *p* <  0.001). Additionally, mothers of higher parity had higher odds of early initiation compared to mothers who had their first birth.

**Table 2:** Multivariable logistic regression modelling the likelihood of early feeding indicators (*n* = 5622)

	Early breastfeeding		Avoiding prelacteal feeding		Optimal early feeding	
	AOR^**1**^ (95% CI)	***p***-value^**2**^	AOR^**1**^ (95% CI)	***p***-value^**2**^	AOR^**1**^ (95% CI)	***p***-value^**2**^
**Region of residence**						
Khartoum	1.00		1.00		1.00	
Northern	0.57 (0.40,0.82)	0.002	0.74 (0.51,1.07)	0.109	0.57 (0.39,0.83)	0.003
Eastern	1.79 (1.21,2.64)	0.004	2.06 (1.46,2.90)	< 0.001	2.02 (1.44,2.85)	< 0.001
Central	0.86 (0.62,1.20)	0.379	1.13 (0.81,1.58)	0.454	0.95 (0.68,1.33)	0.774
Kordofan	0.61 (0.41,0.88)	0.009	1.68 (1.19,2.36)	0.003	1.06 (0.74,1.52)	0.735
Darfur	0.61 (0.44,0.84)	0.003	2.49 (1.80,3.45)	< 0.001	1.25 (0.91,1.73)	0.167
**Maternal age**						
15–24	1.00		1.00		1.00	
25–34	1.17 (0.97,1.41)	0.106	1.25 (1.04,1.51)	0.020	1.24 (1.03,1.50)	0.027
35+	1.06 (0.87,1.29)	0.551	1.28 (1.02,1.61)	0.035	1.25 (1.03,1.51)	0.024
**Mother’s education**						
None	1.00		1.00		1.00	
Primary	0.91 (0.70,1.17)	0.434	1.17 (0.95,1.43)	0.139	1.03 (0.86,1.23)	0.733
Secondary	1.35 (1.02,1.79)	0.034	1.74 (1.34,2.27)	< 0.001	1.62 (1.30,2.02)	< 0.001
Higher	1.13 (0.74,1.72)	0.484	1.37 (0.93,2.01)	0.107	1.43 (0.99,2.06)	0.057
**Father’s education**						
None	1.00					
Primary	1.10 (0.88,1.37)	0.395				
Secondary	1.29 (0.96,1.74)	0.088				
Higher	1.54 (1.04,2.29)	0.033				
Father not in household	0.84 (0.61,1.15)	0.257				
**Planned pregnancy**						
No			1.00		1.00	
Yes			1.51 (1.18,1.93)	0.001	1.33 (1.09,1.62)	0.005
**Place of birth**						
Home					1.00	
Health facility					0.75 (0.60,0.94)	0.013
**Birth assisted by any skilled attendant**						
No	1.00				1.00	
Yes	1.34 (1.06,1.68)	0.014			1.48 (1.19,1.83)	< 0.001
**Birth by caesarean section**						
No	1.00		1.00		1.00	
Yes	0.22 (0.17,0.27)	< 0.001	0.57 (0.45,0.73)	< 0.001	0.34 (0.25,0.47)	< 0.001
**Child sex**						
Male					1.00	
Female					1.16 (1.02,1.33)	0.028
**Child birth order**						
1st	1.00				1.00	
2nd	1.26 (1.06,1.51)	0.002			1.28 (1.08,1.52)	0.005
3rd or higher	1.22 (0.89,1.69)	0.140			1.36 (1.04,1.77)	0.025
*Hosmer and Lemeshow goodness and fit test p*-value		0.664		0.631		0.639

^1^Adjusted Odds Ratio (AOR), adjusted for other variables in the model; ^2^Wald test p-value

### Avoiding prelacteal feeding

Nearly three-quarter of mothers did report not giving prelacteal feeds to their infants (71.7%; 95% CI 69.6, 73.8) (Table 1). Results from univariate logistic regression showed that region of residence, household wealth quintile, planned pregnancy, place of birth and birth by caesarean section were significant predictors of avoiding prelacteal feeding (Supplementary Table S1).

After adjusting for other covariates, region of residence, mother’s age and education, planned pregnancy, and birth by caesarean section were significant factors associated with avoiding prelacteal feeding practice in Sudan (Table 2).

Compared to mothers who lived in Khartoum, the adjusted odds of avoiding prelacteal feeding in the first three days after birth was generally higher in almost all regions with significant odds ratio in Kordofan (AOR 1.68; 95% CI 1.19, 2.36; *p* = 0.003), Eastern (AOR 2.06; 95% CI 1.46, 2.90; *p* <  0.001) and Darfur (AOR 2.49; 95% CI 1.80, 3.45; *p* <  0.001) regions. Maternal age and education up to secondary level were positively associated with avoiding prelacteal feeding. The adjusted odds of avoiding prelacteal feeding was lower among mothers who gave birth by caesarean section compared to those who gave birth by vaginal delivery (AOR 0.57; 95% CI 0.45, 0.73; *p* <  0.001). Likewise, the adjusted odds of avoiding prelacteal feeding was 1.51 times higher for mothers who planned pregnancy of their last child compared to those who did not (AOR 1.51; 95% CI 1.18, 1.93; *p* = 0.001).

### Optimal early feeding

**Fig. 1:**
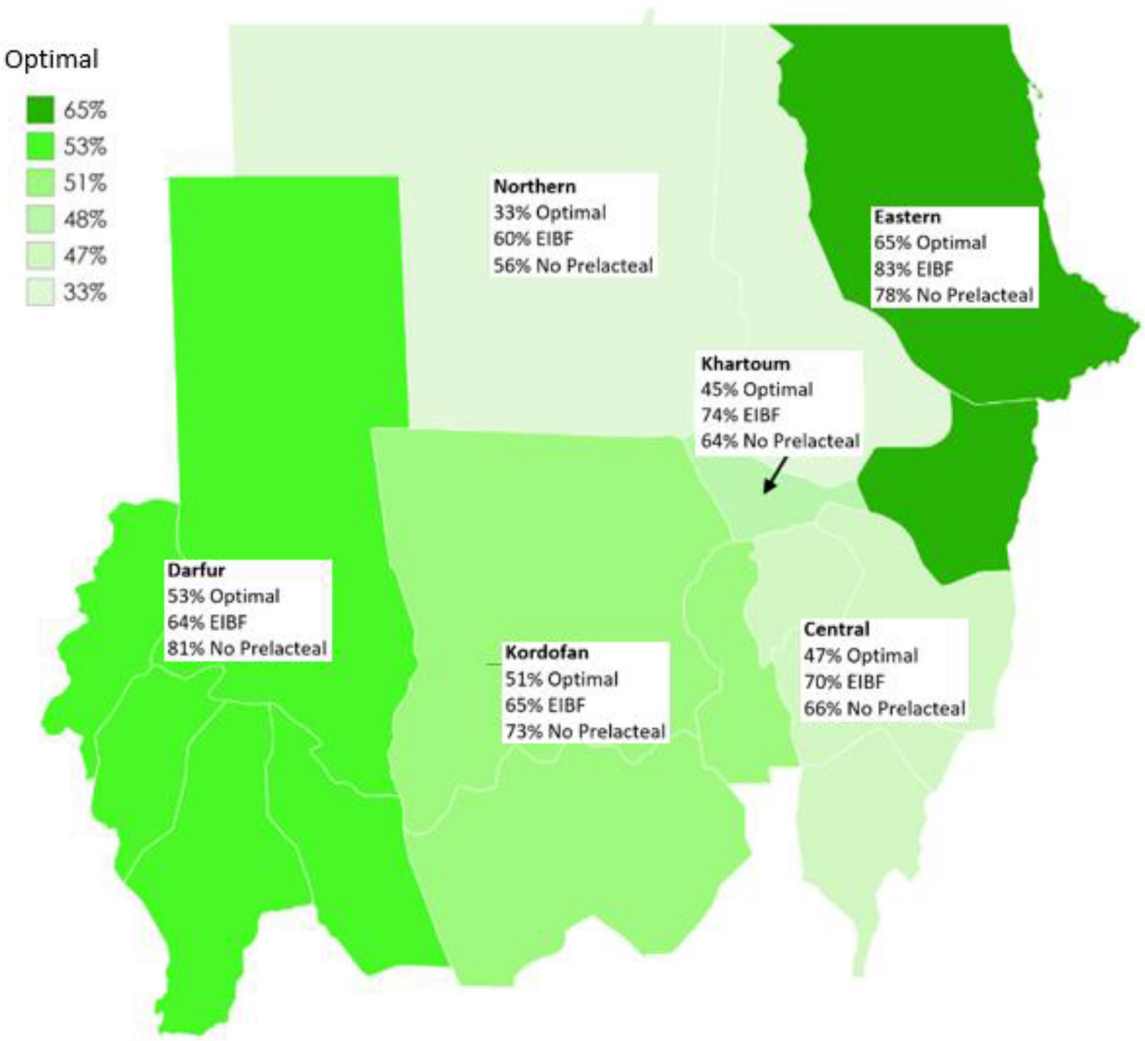
Prevalence of early feeding practices by region of residence

In this study, about half the children had optimal early feeding, i.e. had both early initiation within one hour of birth and did not have any prelacteal feeding in the first three days after birth (51%; 95% CI 48.9, 53.1) (Table 1). Eighteen percent of infants had early initiation within one hour of birth but received prelacteal feeding in the first three days after birth, and 20% of infants did not receive any prelacteal feeding but were not put to the breast within one hour of birth. Optimal early feeding varied by region (Fig. 1), ranging from 33% in the Northern region to 65% in the Eastern region. Univariate logistic regression indicated that optimal feeding was significantly associated with region of residence, wealth, maternal age, planned pregnancy, place of birth, birth by caesarean section, sex of the child, and birth order (Supplementary Table S1).

Results from multivariable logistic analysis (Table 2) revealed that region of residence, mother’s age and education, planned pregnancy, place of birth, birth assisted by a skilled attendant, birth by caesarean section, sex of the child, and birth order were significant predictors of optimal early feeding practice.

The adjusted odds of optimal early feeding practice was two times higher in the Eastern region (AOR 2.02; 95% CI 1.44, 2.85; *p* <  0.001) and 43% lower in Northern region (AOR 0.57; 95% CI 0.39, 0.83; *p* = 0.003) when compared to Khartoum. Maternal age was significantly associated with optimal feeding with older women having about 25% higher odds of practicing optimal feeding related to the youngest women aged 15–24 years (e.g. for women aged 34+ years AOR 1.25; 95% CI 1.03, 1.51; *p* = 0.024). Mothers who had secondary or higher education had a 62% (AOR 1.62; 95% CI 1.30, 2.02; *p* <  0.001) and 43% (AOR 1.43; 95% CI 0.99, 2.06; *p* = 0.057) increased odds of optimal early feeding respectively compared to those with no education. Mothers with a planned pregnancy had 1.33 times higher odds to optimally feed their infants (AOR 1.33; 95% CI 1.09, 1.62; *p* = 0.005). In comparison to home births, children who were given birth in a health facility were less likely to be fed optimally (AOR 0.75; 95% CI 0.60, 0.94; *p* = 0.013). Compared to vaginal delivery, caesarean section was significantly associated with a much lower odds of optimal feeding (AOR 0.34; 95% CI 0.25, 0.47; *p* <  0.001). On the contrary, skilled attendance at birth increased the odds of optimal early feeding (AOR 1.48; 95% CI 1.19, 1.83; *p* <  0.001). Sex of child was also significantly related to optimal feeding practice in this study with 16% higher odds of optimal feeding for mothers who had female vs male children (AOR 1.16; 95% CI 1.02, 1.33; *p* = 0.028). Moreover, compared to mothers with 1st.

birth, the odds of optimal early feeding among mothers with 2nd and 3rd or higher birth order were (AOR 1.28; 95% CI 1.08, 1.52; *p* = 0.005) and (AOR 1.36; 95% CI 1.04, 1.77; *p* = 0.025), respectively.

## Discussion

Achieving a 50% exclusive breastfeeding rate during the first six months of life is one of the 2025 WHO global targets [32]. Optimal early feeding practice, defined as both early initiation of breastfeeding within one hour of birth and no prelacteal feeding in the first three days after birth, will contribute to the success of exclusive breastfeeding. This aim of this study was to determine the prevalence and associated factors of early initiation within one hour of birth, no prelacteal feeding and optimal early feeding in Sudan using nationally representative data.

The prevalence of early initiation in this study (69%) was relatively similar to Sudan national estimate in 2010 [33] and levels of Namibia and Zimbabwe [34] and Oman and Iran [8, 35]. The prevalence of early initiation in this study is higher than the WHO target of 50% by the year 2025 [32]. It is also higher than the overall level in 30 SSA countries [16, 36], neighbouring country South Sudan and most MENA countries like Egypt and Tunisia (40%). On the contrary, the early initiation prevalence in this study is lower than in some Eastern African countries such as Malawi and Rwanda [34].

The results report that 72% of infants did not receive prelacteal feeds within the first three days of birth. Avoiding these feeds promotes the implementation of optimal breastfeeding practices. The observed avoidance prevalence in this study is higher than the overall level of 30 Sub Saharan African (SSA) countries [16, 36] and higher than prevalences from studies conducted in neighbouring country South Sudan [37] and other developing countries such as Ethiopia [38] and other SSA countries [14].

The results of this study found that despite the relatively high percentages of women breastfeeding their infants in the first hour after birth and refraining from giving their infants any prelacteal feeds in the first three days after birth, only half of them adhere to both good practices. This level of optimal early feeding is higher than overall levels reported by Oakley et al. (28%) but lower than several East African countries such as Malawi (93.2%), Mozambique (74.5%), Burundi (71.3%) and Rwanda (66.2%) [16]. Variations between-country may be due to differences in knowledge of the advantages of early initiation, social and cultural practice related to prelacteal feeding, differences in advice from the elderly and significant family members, religious beliefs, as well as variations in the health system and the support offered by health professionals and birth attendants [12, 39, 40]. Household wealth may not be an explanation as originally hypothesized as wealth index was not significantly associated with any of the outcomes in this study similar to a study from Tanzania [41, 42].

The study found considerable regional variations in early breastfeeding indicators. Sudan consists of diverse ethnic tribes clustering in different regions with the capital Khartoum generally representing the whole country ethnic structure. Living situations across communities vary with access to health care facilities, media and health information. Previous studies conducted in different regions of Sudan also reported varying prevalence in breastfeeding behaviour [17, 43].

After adjustment, this study found the following common factors as significant factors of the three early feeding practices indicators in Sudan: region, maternal education and birth by caesarean section. Maternal age was a significant factor for both no prelacteal and optimal feeding indicators. This study result of no prelacteal feeding was consistent with those reported in other studies [14, 44]. Compared to mothers with no education, this study found an increasing trend in the odds of early initiation, no prelacteal feeding and optimal feeding with maternal education up to secondary level. These findings are consistent with those from previous studies [14, 45, 46]. However, two studies done in Kassala State [17] and South Sudan [47] did not find maternal education to be a significant factor associated with early initiation. The reason may be attributed to the fact that these studies were either community or hospital-based studies with smaller sample sizes than this national study.

Caesarean section has been consistently associated with lower odds of early initiation and a higher prevalence of prelacteal feeding [7, 8, 14, 17, 47, 48]. Caesarean section may prevent immediate contact between mother and baby and of breastfeeding due to post anaesthesia or postoperative effects, thus encouraging prelacteal feeding [48]. Besides, due to the pain associated with surgery [49] and concern that antibiotics and other medications received after surgery could harm the infants, mothers may be uncomfortable to start breastfeeding immediately after birth [29]. Although less than 10% of mothers in Sudan give birth by caesarean sections, the associated poor early breastfeeding indicators should still be addressed.

The finding that birth assisted by a skilled attendant is a significant factor associated with early initiation and optimal feeding practices is consistent with findings reported by Oakley et al. [16]. The authors of this study suggested that wealthier women tend to give birth at health facilities and thus have their deliveries assisted by skilled attendants. This is consistent with this study, where only 48% of the women in the lowest wealth quintile were assisted by skilled attendant compared to 100% in the highest quintile [33].

This study found no association between early initiation and no prelacteal feeding indicators with place of birth (less than 2% of the births in this study were in private health facilities). This is in contrast to the overall findings reported by Bergamaschi et al. [36] associating better outcome of early initiation and no prelacteal feeding indicators with health facilities. Nonetheless, Bergamaschi et al. identified several countries in SSA, like Gabon and Liberia, favouring home deliveries to health facility deliveries regarding the early initiation indicator. There is little evidence in the literature associating home deliveries with better early breastfeeding indicators. The reason of observing this association in Sudan may be due to deficiencies in proper health education and breastfeeding promotion to mothers who give birth in health facilities particularly with the shortage of implementation of BFHI in the country [24]. Another reason may be the involvement of non-profit organisations like the Sudanese Association for Breastfeeding Action (SABA) in community awareness campaigns training midwives on optimal breastfeeding practices.

Almost three-quarters of births are delivered at home in Sudan, particularly in rural areas comprising three-quarters of the population (Table 1). Improved early feeding indicators may be due several factors not limited to social and cultural factors, better knowledge and promotion of breastfeeding among midwives who mainly deliver mothers at homes, increased use of bottle feeding in urban areas (10.6%) than in rural (6.2%) [27] and utilisation of breastfeeding as a natural form of family planning in rural areas [12]. There is a need for further research on the reasons for having better early feeding practices in Sudan among mothers who give birth at homes compared to those who give birth at health facilities.

### Implications

About two of three Sudanese mothers initiate breastfeeding early after childbirth. A similar number of Sudanese mothers avoid prelacteal feeding in the first three days after childbirth. Nonetheless, only half of these mothers follow both practices at the same time. The National Strategy for Infant and Young Child Feeding (IYCF) 2015–2024 established by the national nutrition program of Sudan Federal Ministry of Health address the challenges of the significant suboptimal IYCF practices in Sudan. The strategy calls for improvement of the implementation of BFHI and improvement of knowledge and skills of health service providers. This study provides evidence-based information to benefit the implementation of the strategy.

### Strengths and limitations

This research is the first in Sudan to analyse a nationally representative sample to explore early feeding indicators. The data set was nationally representative with low levels of missing data. Another strength in this study is the use of a new optimal feeding indicator which captures two important early feeding practices (early initiation and avoidance of prelacteal feeding). The study had some limitations that should be considered while interpreting the results. Firstly, although the MICS data provided the opportunity to control for several confounding factors, it did not allow to control directly for other important dimensions like social and cultural factors like the importance of advice from grandmothers and ethnic beliefs on infant early feeding [12, 40]. Nonetheless, apart from the diverse capital city, considering the cultural diversity in Sudan, this aspect may have been implicitly controlled for when assessing regional differences. Secondly, as with cross-sectional studies, this study does not allow inferring causality. Thirdly, recall bias may be potential when mothers recall information on early feeding practices of their children that happened up to two years preceding the survey. Lastly, the way in which missing values in the outcomes were handled may produce slight underestimation in the early breastfeeding indicator and overestimation in the avoidance of prelacteal feeding indicator.

## Conclusions

Only half of Sudanese infants received optimal early feeding practice, with notable differences between regions in the country. Early breastfeeding practices in Sudan are associated with various community levels, maternal and child factors. The findings suggest the need to develop breastfeeding promotion programs considering regional variations in the country. These programs should be targeted toward mothers giving birth by caesarean section and their health providers. The findings also suggest improving the knowledge and skills of health providers working at health facilities on IYCF education, and community-based health workers like traditional birth attendants to providing early postnatal visits and education to women who give birth at home. Further studies should be conducted to address the possible reasons for having better early breastfeeding indicators in mothers who give birth at home.

## Supplementary information

**Additional file 1: **Supplementary **Table S1**: Univariate logistic regression modelling the likelihood of early feeding indicators (*n* = 5622).

## Data Availability

The dataset for this study is freely available from the MICS website (http://mics.unicef.org/). Permission to use and analyse the data was obtained through a request to UNICEF through their website.

## Abbreviations

MICS: Sudan Multiple Indicator Cluster Survey
WHO: World Health Organization
BFHI: Baby-Friendly Hospital Initiative
SSA: Sub-Saharan African
AOR: Adjusted odds ratio
