# Supplementary material for: Early feeding practices and associated factors in Sudan: a cross-sectional analysis from multiple Indicator cluster survey
Source: Int Breastfeed J. 2020 May 14;15:41. doi: 10.1186/s13006-020-00288-7 (PMC7227029; doi:10.1186/s13006-020-00288-7)
Supplement: Supplementary file 1 — Additional file 1: Supplementary Table S1: Univariate logistic regression modelling the likelihood of early feeding indicators (n = 5622). [file 13006_2020_288_MOESM1_ESM.docx]

Supplementary Table S1: Univariate logistic regression modelling the likelihood of early feeding indicators (n=5,622)

|  | **Early breastfeeding** | |  | **Avoiding prelacteal feeding** | |  | **Optimal early feeding** | |
| --- | --- | --- | --- | --- | --- | --- | --- | --- |
|  | **OR (95% CI)** | **p-value^1^** |  | **OR (95% CI)** | **p-value^1^** |  | **OR (95% CI)** | **p-value^1^** |
| **Community-level variables** |  |  |  |  |  |  |  |  |
| **Area of residence** |  | 0.155 |  |  | 0.266 |  |  | 0.660 |
| Urban | 1.00 |  |  | 1.00 |  |  | 1.00 |  |
| Rural | 0.86 (0.71,1.06) |  |  | 1.13 (0.91,1.39) |  |  | 1.04 (0.87,1.24) |  |
|  |  |  |  |  |  |  |  |  |
| **Region of residence** |  | <0.001 |  |  | <0.001 |  |  | <0.001 |
| Khartoum | 1.00 |  |  | 1.00 |  |  | 1.00 |  |
| Northern | 0.53 (0.38,0.76) |  |  | 0.72 (0.51,1.02) |  |  | 0.55 (0.38,0.78) |  |
| Eastern | 1.78 (1.22,2.59) |  |  | 1.96 (1.43,2.68) |  |  | 2.04 (1.48,2.79) |  |
| Central | 0.83 (0.60,1.15) |  |  | 1.08 (0.78,1.49) |  |  | 0.98 (0.72,1.34) |  |
| Kordofan | 0.65 (0.45,0.94) |  |  | 1.55 (1.11,2.16) |  |  | 1.13 (0.81,1.58) |  |
| Darfur | 0.62 (0.46,0.84) |  |  | 2.31 (1.67,3.18) |  |  | 1.25 (0.94,1.66) |  |
|  |  |  |  |  |  |  |  |  |
| **Household-level variables** |  |  |  |  |  |  |  |  |
| **Wealth index quintile** |  | 0.004 |  |  | <0.001 |  |  | 0.012 |
| Poorest | 1.00 |  |  | 1.00 |  |  | 1.00 |  |
| Second | 1.41 (1.11,1.79) |  |  | 1.10 (0.83,1.45) |  |  | 1.21 (0.97,1.50) |  |
| Middle | 1.66 (1.27,2.17) |  |  | 0.72 (0.54,0.97) |  |  | 1.05 (0.83,1.35) |  |
| Fourth | 1.31 (1.00,1.71) |  |  | 0.60 (0.43,0.85) |  |  | 0.79 (0.59,1.05) |  |
| Richest | 1.51 (1.12,2.04) |  |  | 0.65 (0.47,0.90) |  |  | 0.89 (0.68,1.17) |  |
|  |  |  |  |  |  |  |  |  |
| **Parental variables** |  |  |  |  |  |  |  |  |
| **Maternal age** |  | 0.059 |  |  | 0.219 |  |  | 0.029 |
| 15-24 | 1.00 |  |  | 1.00 |  |  | 1.00 |  |
| 25-34 | 1.22 (1.03,1.43) |  |  | 1.17 (0.97,1.42) |  |  | 1.25 (1.05,1.50) |  |
| 35+ | 1.07 (0.90,1.27) |  |  | 1.16 (0.93,1.43) |  |  | 1.20 (1.00,1.43) |  |
|  |  |  |  |  |  |  |  |  |
| **Maternal age at marriage** |  | 0.117 |  |  | 0.437 |  |  | 0.092 |
| <15 | 1.00 |  |  | 1.00 |  |  | 1.00 |  |
| 15-24 | 1.24 (1.00,1.53) |  |  | 1.18 (0.92,1.51) |  |  | 1.26 (1.02,1.54) |  |
| 25+ | 1.27 (0.92,1.76) |  |  | 1.17 (0.81,1.69) |  |  | 1.25 (0.95,1.64) |  |
| **Mother's education** |  | 0.122 |  |  | 0.221 |  |  | 0.229 |
| None | 1.00 |  |  | 1.00 |  |  | 1.00 |  |
| Primary | 0.98 (0.79,1.22) |  |  | 0.92 (0.75,1.13) |  |  | 0.94 (0.79,1.12) |  |
| Secondary | 1.28 (1.01,1.62) |  |  | 1.13 (0.88,1.45) |  |  | 1.15 (0.93,1.42) |  |
| Higher | 1.04 (0.73,1.49) |  |  | 0.79 (0.56,1.13) |  |  | 0.86 (0.62,1.17) |  |
|  |  |  |  |  |  |  |  |  |
| **Father's education** |  | 0.004 |  |  | 0.836 |  |  | 0.052 |
| None | 1.00 |  |  | 1.00 |  |  | 1.00 |  |
| Primary | 1.07 (0.89,1.29) |  |  | 0.98 (0.79,1.23) |  |  | 1.05 (0.87,1.27) |  |
| Secondary | 1.23 (0.97,1.57) |  |  | 0.99 (0.78,1.25) |  |  | 1.10 (0.88,1.38) |  |
| Higher | 1.45 (1.03,2.03) |  |  | 0.85 (0.60,1.21) |  |  | 1.10 (0.82,1.49) |  |
| Father not in household | 0.80 (0.60,1.05) |  |  | 1.06 (0.79,1.41) |  |  | 0.77 (0.60,0.99) |  |
|  |  |  |  |  |  |  |  |  |
| **Planned pregnancy** |  | 0.382 |  |  | <0.001 |  |  | 0.006 |
| No | 1.00 |  |  | 1.00 |  |  | 1.00 |  |
| Yes | 1.10 (0.88,1.38) |  |  | 1.50 (1.20,1.88) |  |  | 1.29 (1.07,1.54) |  |
|  |  |  |  |  |  |  |  |  |
| **Maternal health care services** |  |  |  |  |  |  |  |  |
| **Number of times received antenatal care** |  | 0.306 |  |  | 0.649 |  |  | 0.621 |
| 0 | 1.00 |  |  | 1.00 |  |  | 1.00 |  |
| 1-3 | 1.20 (0.95,1.52) |  |  | 0.91 (0.69,1.21) |  |  | 0.94 (0.76,1.16) |  |
| 4+ | 1.09 (0.87,1.37) |  |  | 0.88 (0.68,1.15) |  |  | 0.90 (0.73,1.11) |  |
|  |  |  |  |  |  |  |  |  |
| **Place of birth** |  | <0.001 |  |  | <0.001 |  |  | <0.001 |
| Home | 1.00 |  |  | 1.00 |  |  | 1.00 |  |
| Health facility | 0.72 (0.60,0.85) |  |  | 0.68 (0.56,0.82) |  |  | 0.62 (0.53,0.73) |  |
|  |  |  |  |  |  |  |  |  |
| **Birth assisted by any skilled attendant** |  | 0.003 |  |  | 0.754 |  |  | 0.141 |
| No | 1.00 |  |  | 1.00 |  |  | 1.00 |  |
| Yes | 1.35 (1.11,1.65) |  |  | 0.96 (0.77,1.21) |  |  | 1.15 (0.95,1.39) |  |
|  |  |  |  |  |  |  |  |  |
| **Birth by caesarean section** |  | <0.001 |  |  | <0.001 |  |  | <0.001 |
| No | 1.00 |  |  | 1.00 |  |  | 1.00 |  |
| Yes | 0.31 (0.24,0.39) |  |  | 0.52 (0.41,0.66) |  |  | 0.32 (0.24,0.42) |  |
|  |  |  |  |  |  |  |  |  |
| **Child variables** |  |  |  |  |  |  |  |  |
| **Sex** |  | 0.221 |  |  | 0.080 |  |  | 0.040 |
| Male | 1.00 |  |  | 1.00 |  |  | 1.00 |  |
| Female | 1.09 (0.95,1.24) |  |  | 1.13 (0.99,1.29) |  |  | 1.14 (1.01,1.30) |  |
|  |  |  |  |  |  |  |  |  |
| **Birth order** |  | 0.022 |  |  | 0.264 |  |  | 0.008 |
| 1st | 1.00 |  |  | 1.00 |  |  | 1.00 |  |
| 2nd | 1.25 (1.07,1.47) |  |  | 1.14 (0.97,1.33) |  |  | 1.27 (1.09,1.49) |  |
| 3rd or higher | 1.20 (0.90,1.62) |  |  | 1.13 (0.87,1.46) |  |  | 1.31 (1.02,1.68) |  |
|  |  |  |  |  |  |  |  |  |
| **Perceived size at birth** |  | 0.085 |  |  | 0.325 |  |  | 0.296 |
| Larger than average | 1.00 |  |  | 1.00 |  |  | 1.00 |  |
| Average | 1.26 (1.01,1.58) |  |  | 0.97 (0.76,1.24) |  |  | 1.16 (0.93,1.46) |  |
| Smaller than average | 1.11 (0.87,1.43) |  |  | 0.86 (0.66,1.13) |  |  | 1.06 (0.82,1.36) |  |
| *^1^Likelihood ratio test p-values* |  |  |  |  |  |  |  |  |
